# RDL mutations predict multiple insecticide resistance in *Anopheles sinensis* in Guangxi, China

**DOI:** 10.1186/s12936-017-2133-0

**Published:** 2017-11-28

**Authors:** Chan Yang, Zushi Huang, Mei Li, Xiangyang Feng, Xinghui Qiu

**Affiliations:** 1 State Key Laboratory of Integrated Management of Pest Insects and Rodents, Institute of Zoology, Chinese Academy of Sciences, Beijing, 100101 China; 2 University of Chinese Academy of Sciences, Beijing, 100049 China; 3 Guangxi Zhuang Autonomous Region Centre for Diseases Control and Prevention, Nanning, 530028 China

**Keywords:** *Anopheles sinensis*, Haplotype, Genealogical analysis, Guangxi Zhuang Autonomous Region, Gamma-aminobutyric acid gated chloride channel

## Abstract

**Background:**

*Anopheles sinensis* is a major vector of malaria in China. The gamma-aminobutyric acid (GABA)-gated chloride channel, encoded by the *RDL* (Resistant to dieldrin) gene, is the important target for insecticides of widely varied structures. The use of various insecticides in agriculture and vector control has inevitably led to the development of insecticide resistance, which may reduce the control effectiveness. Therefore, it is important to investigate the presence and distribution frequency of the resistance related mutation(s) in *An. sinensis* RDL to predict resistance to both the withdrawn cyclodienes (e.g. dieldrin) and currently used insecticides, such as fipronil.

**Methods:**

Two hundred and forty adults of *An. sinensis* collected from nine locations across Guangxi Zhuang Autonomous Region were used. Two fragments of *An. sinensis RDL* (*AsRDL*) gene, covering the putative insecticide resistance related sites, were sequenced respectively. The haplotypes of each individual were reconstructed by the PHASE2.1 software, and confirmed by clone sequencing. The phylogenetic tree was built using maximum-likelihood and Bayesian inference methods. Genealogical relations among different haplotypes were also analysed using Network 5.0.

**Results:**

The coding region of *AsRDL* gene was 1674 bp long, encoding a protein of 557 amino acids. *AsRDL* had 98.0% amino acid identity to that from *Anopheles funestus*, and shared common structural features of Cys-loop ligand-gated ion channels. Three resistance-related amino acid substitutions (A296S, V327I and T345S) were detected in all the nine populations of *An. sinensis* in Guangxi, with the 296S mutation being the most abundant (77–100%), followed by 345S (22–47%) and 327I (8–60%). 38 *AsRDL* haplotypes were identified from 240 individuals at frequencies ranging from 0.2 to 34.8%. Genealogical analysis suggested multiple origins of the 345S mutation in *AsRDL*.

**Conclusions:**

The near fixation of the 296S mutation and the occurrence of the 327I and 345S mutations in addition to 296S, in all the nine tested *An. sinensis* populations in Guangxi, strongly indicate a risk of multiple insecticide resistance. The haplotype diversity plus genetic heterogeneities in the geographical distribution, and multiple origins of *AsRDL* alleles call for a location-customized strategy for monitoring and management of insecticide resistance.

## Background

Guangxi Zhuang Autonomous Region was once a malaria-endemic region with more than 5 million malaria patients per year being recorded before 1949 [1]. After continued efforts, particularly the “National Malaria Control Programme”, the “Basically Eliminating Malaria” and the “Action Plan of Malaria Elimination (2010–2020)” [2, 3], the malaria burden has been substantially reduced in Guangxi [4]. However, the risk of malaria re-emergence remains, because many imported malaria cases have been reported due to ever increasing cross-border population migration, and the natural environment in Guangxi, consisting of rice fields, provides many mosquito breeding sites [3–6].

Chemical control of vectors has played an important role in malaria control and elimination [7]. In China, *Anopheles sinensis* is a major vector of malaria. The use of insecticides in the control of *An. sinensis* itself, and in agriculture, has inevitably led to increasing insecticide resistance in Chinese *An. sinensis* [8–10]. The development of insecticide resistance can reduce the effectiveness of vector control, therefore, understanding the levels of resistance and mechanisms responsible for this resistance is a core concept to effectively manage *An. sinensis.* However, the insecticide resistance issue in *An. sinensis* has so far received little attention. Although the distribution and frequency of insecticide resistance-conferring mutations in the acetylcholinesterase and voltage gated sodium channel were determined in *An. sinensis* collected extensively across Guangxi in previous studies [11, 12], other questions related to insecticide resistance remain to be answered.

The insect gamma-aminobutyric acid (GABA) receptor subunit encoded by the *RDL* (Resistant to dieldrin) gene plays a central role in neuronal signaling and is involved in various processes [13]. RDL has been the target for insecticides of various chemical structures such as cyclodienes and fipronil [14]. RDL is also a potential secondary target for neonicotinoids and pyrethroids [13]. Previous studies have documented that mutations in RDL are associated with insecticide resistance in multiple insect species. For example, the replacement of a single alanine (Ala301 in *Drosophila*) at the start of transmembrane segment M2 with either serine or glycine makes the *Drosophila* strains resistant to dieldrin [15]. Similarly, the A296S substitution (analogous to position 301 in *Drosophila melanogaster*) was associated with dieldrin resistance in mosquitoes such as *Anopheles arabiensis*, *Anopheles funestus, Anopheles stephensi, Aedes aegypti, Aedes albopictus* and *Culex pipiens quinquefasciatus* [16–19]. Another replacement of the equivalent residue (A301N) was observed in the fipronil-resistant planthopper *Laodelphax striatellus* [20, 21]. Besides mutation at residue 296, a substitution (V327I) was reported on a background of 296S in dieldrin-resistant *An. funestus* [18]. In addition, two different substitutions at residue 350 (T350M and T350S) were observed in *Drosophila* linked with 301G [22] and 301S [23], respectively. Recently, a second RDL mutation (R300Q) in combination with A302S was found to be associated with much higher levels of fipronil resistance (237-fold) in *Nilaparvata lugens* [24].

Until now, there is no published report about RDL in the malaria vector *An. sinensis*. In this study, the *RDL* gene in *An. sinensis* (*AsRDL*) was identified, and the naturally occurring genetic mutations in *AsRDL* and their geographical distribution in Guangxi were investigated, in the hope of predicting their both historical role in cyclodiene (e.g. dieldrin) resistance and potential influence in conferring resistance to currently used insecticides such as fipronil.

## Methods

### Samples

*Anopheles sinensis* adults used in the study were caught by light trap (wave length 365 nm) from July to September in 2014, around farmers’ houses at different geographical locations across Guangxi. The sampling sites were part of the malaria vector monitoring sites that were set up partly because there were imported malaria cases observed since 2010 in these regions. A brief description of the sampling sites was provided previously [12].

*Anopheles sinensis* individual mosquitoes were morphologically identified [25], and put into 100-μl Eppendorf tubes containing 100% ethanol solution at 4 °C. The accuracy of morphological classification was confirmed by molecular detection of ten randomly selected adults from each population using the rDNA-ITS2 method [26].

### Sequencing of the full length *RDL* coding sequence from *Anopheles sinensis*

Total RNA was extracted from ten adults of a laboratory strain of *An. sinensis* [27] by using TRIzol (Invitrogen, CA, USA) according to the manufacturer’s protocol. First-strand cDNA was synthesized from total RNA (1 µg) using PrimeScript First Strand Synthesis Kit according to the manufacturer’s instructions (Takara, Dalian, China). The full length open reading frame sequence of the GABA-gated chloride channel gene was amplified by PCR using the primers (AsRDLfullcd-F:ATGTCGCTAACCATCGAAGTTCCGC; AsRDLfullcd-R: TTACTTATCCTCACCGAGCAGCA) commercially synthesized by Invitrogen (China). The PCR mixture (50 μl) consisted of 2 μl cDNA template, 1 μl PrimeStar^®^ GXL (Takara), 10 μl 5× Buffer, 4 μl 2.5 μM dNTPs, 1 μl 10 mM each primer, and 31 μl ddH_2_O. The thermal cycling profile consisted of an initial step of denaturation at 95 °C for 3 min; followed by 36 cycles of 98 °C for 10 s, 55 °C for 15 s, 68 °C for 2 min; and a final extension step at 68 °C for 10 min. The PCR products were gel purified (Takara, Dalian, China) and then subcloned into pEasy-T1 and transformed the *Escherichia coli* Trans 5α strain (Transgen, Beijing, China). The nucleotide sequences of *As*-*RDL* gene were identified by direct sequencing of PCR products and/or clone sequencing from two directions.

### PCR amplification of *AsRDL* fragments

The genomic DNA of individual adult mosquitoes was prepared as described elsewhere [11, 28]. A fragment of exon 7 encoding the M2 transmembrane domain region was amplified using primers ASRdl-7F (AGTTTGTACGTTCGATGGGTTA) and ASRdl-7R (CCAGCAGACTGGCAAATACC). Another fragment containing partial intron 7 and exon 8 was obtained using primers ASRdl-8F **(**GCGTGGCACTGATATCGTTT) and ASRdl-8R (CCTTGCCGGGATGTATCAGA). The PCR mixture (20 μl) consisted of 5 μl DNA template, 0.5 μl Takara Taq Polymerase, 2 μl 10× Taq Buffer, 2 μl 2.5 μM dNTPs, 0.4 μl 10 mM each primer, and 9.7 μl ddH_2_O. The reaction program was 95 °C for 5 min, followed by 38 cycles each with 95 °C for 30 s, 55 °C for 30 s, 72 °C for 30 s, and by a final extension of 10 min at 72 °C. PCR products were gel-purified and sequenced using ASRdl-7F and ASRdl-8R, respectively (BGI, China).

### Haplotype reconstruction and confirmation

Genotype data were carefully inspected by using Chromas 2.01 (Technelysium Pty Ltd, Australia). *AsRDL* haplotypes were reconstructed by software PHASE2.1 [29]. The presence (and accuracy) of the haplotypes that were reconstructed by PHASE in heterozygotes was confirmed by sequencing of three to five clones.

### Genealogical analysis

The *AsRDL* haplotypes were used to build the phylogenetic topologies by maximum-likelihood (ML) and Bayesian inference (BI) methods. The best-fitting substitution model was determined under the Bayesian information criteria (BIC) using jModelTest 2.1.1 [30]. The best-fitting model of nucleotide substitution for *AsRDL* fragment was TVM+I. PhyML 3.0 was used to perform ML analyses [31]. The non-parametric bootstrap with 1000 replicates was used to estimate the support for each internal branch of the ML phylogeny. The program MrBayes 3.2.1 was used for Bayesian phylogenetic inference [32]. Markov chains were run for 10^7^ generations with two independent runs, and trees were sampled every 1000 generations. The initial 25% of trees were discarded as burn-in, and the remaining trees (15,002) were used to estimate the majority-rule consensus tree and the Bayesian posterior probabilities (BPP). Genealogical relations among different haplotypes were also analysed using Network 5.0 [33].

## Results

### Identification of *AsRDL* gene

**Fig. 1:**
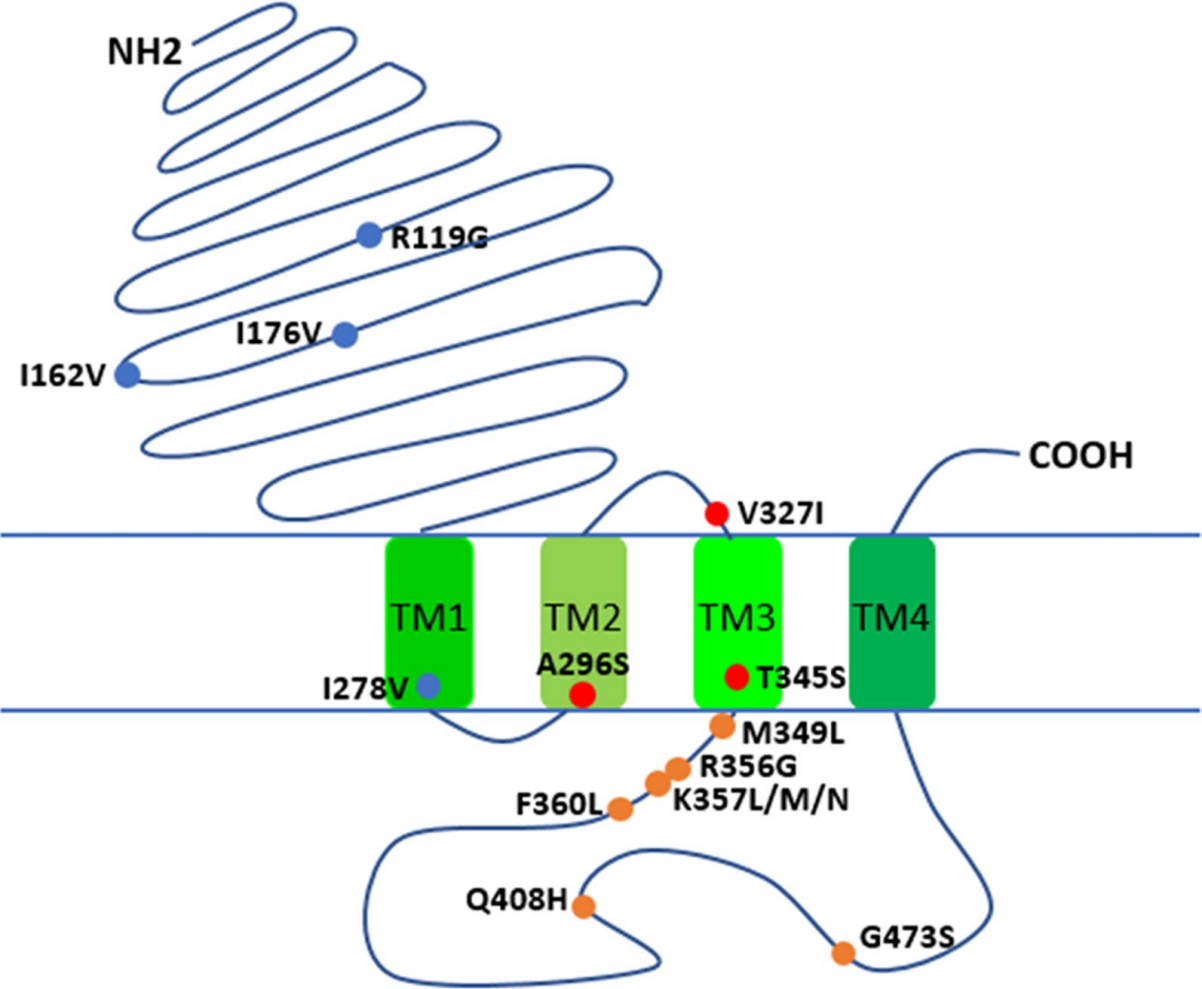
Schematic representation of polymorphisms in AsRDL monomer. The substitutions indicated by blue dots (R119G, I162V, I176V, I278V) are identified in a laboratory strain. The insecticide resistance-related mutations (296S, 327I, 345S) are marked by red dots. The polymorphisms indicated by orange dots are detected in field mosquitoes (M349L, R356G, K357L/M/N, F360L, Q408H, G473S)

**Fig. 2:**
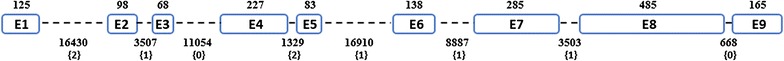
Exon/intron organization of the *AsRDL* gene. E represents exon and dash line represents intron. The numbers above the box or below the dash line represent the length (base pair) of different parts of *AsRDL* gene. The intron phase is indicated using braced numbers: 0 means intron splicing between codons, 1 means intron splicing between the first and second nucleotides of a codon, and 2 means intron splicing between the second and third nucleotides of a codon

The coding region of *AsRDL* gene was 1674 bp long, encoding a protein of 557 amino acids. Four non-synonymous variations that result in amino acid substitutions (R119G, I162V, I176V, I278V) were identified in cDNA sequences (Fig. 1). Mapping the coding region to the genomic sequence (KE525297.1**)** showed that the *AsRDL* gene consists of nine exons and eight introns (Fig. 2).

**Fig. 3:**
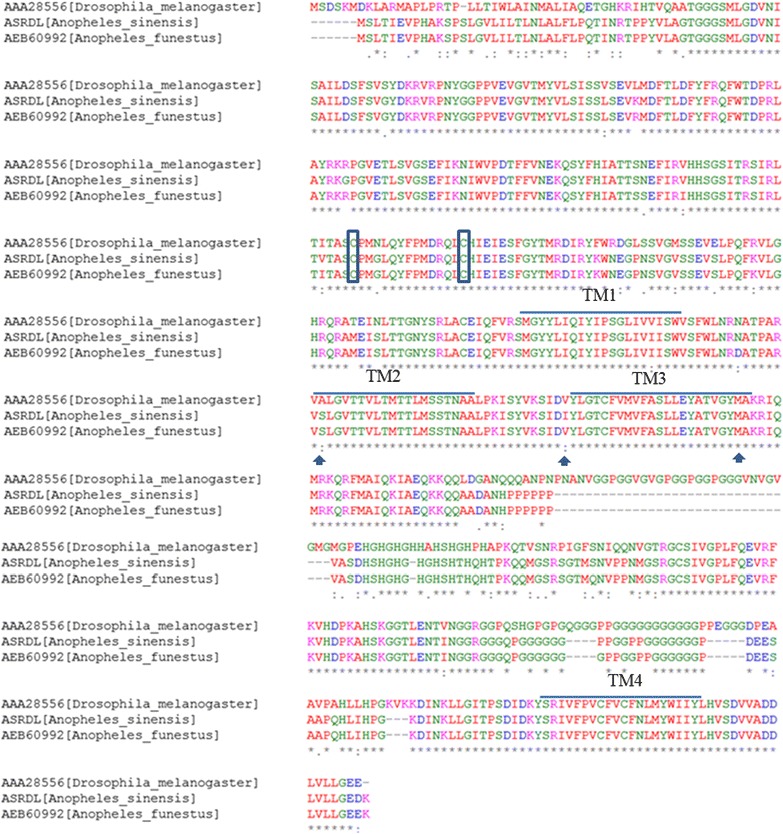
Alignment of amino acid sequences of RDLs from *Anopheles sinensis* (AsRDL), *An. funestus* (AEB60992) and *Drosophila melanogaster* (AAA28556). The asterisks (*) indicate identical amino acid, colons (:) represent conserved substitution and black dots (·) indicate weakly conserved sites. The three insecticide resistance-related residues (296, 327,345) are marked with arrows. The four transmembrane regions (TM1–4) are indicated by a straight line. The two cysteines forming the cys-loop are in box

Alignment analysis (Fig. 3) showed that AsRDL was highly similar to that from *Anopheles funestus* (98.0% amino acid identity), followed by *Anopheles gambiae* (97.9%). A lower identity (77.3%) was determined when compared with *Drosophila melanogaster* RDL. AsRDL had the common structural features shared by known Cys-loop ligand-gated ion channels (LGICs), including the well-conserved dicysteine-loop structure, a large extracellular domain and four hydrophobic transmembrane regions (TM 1–4) (Fig. 3).

### Identification of naturally occurring genetic mutations in *AsRDL*

**Fig. 4:**
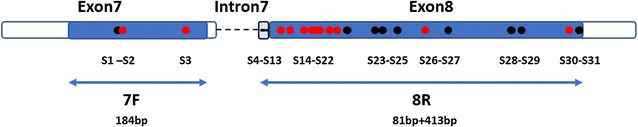
The polymorphic sites (S) identified in the *AsRDL* gene in this study. Non-synonymous sites are indicated by red dots

A 184 bp-in-length sequence of partial exon 7 (namely 7F), and a 494 bp region containing 81 bp in intron 7 and 413 bp in exon 8 (namely 8R), were obtained from 240 individuals, respectively. Three polymorphic sites were observed in 7F (Fig. 4). From the 8R sequences, ten and eighteen polymorphic sites were identified in intron 7 and exon 8, respectively (Fig. 4).

**Fig. 5:**
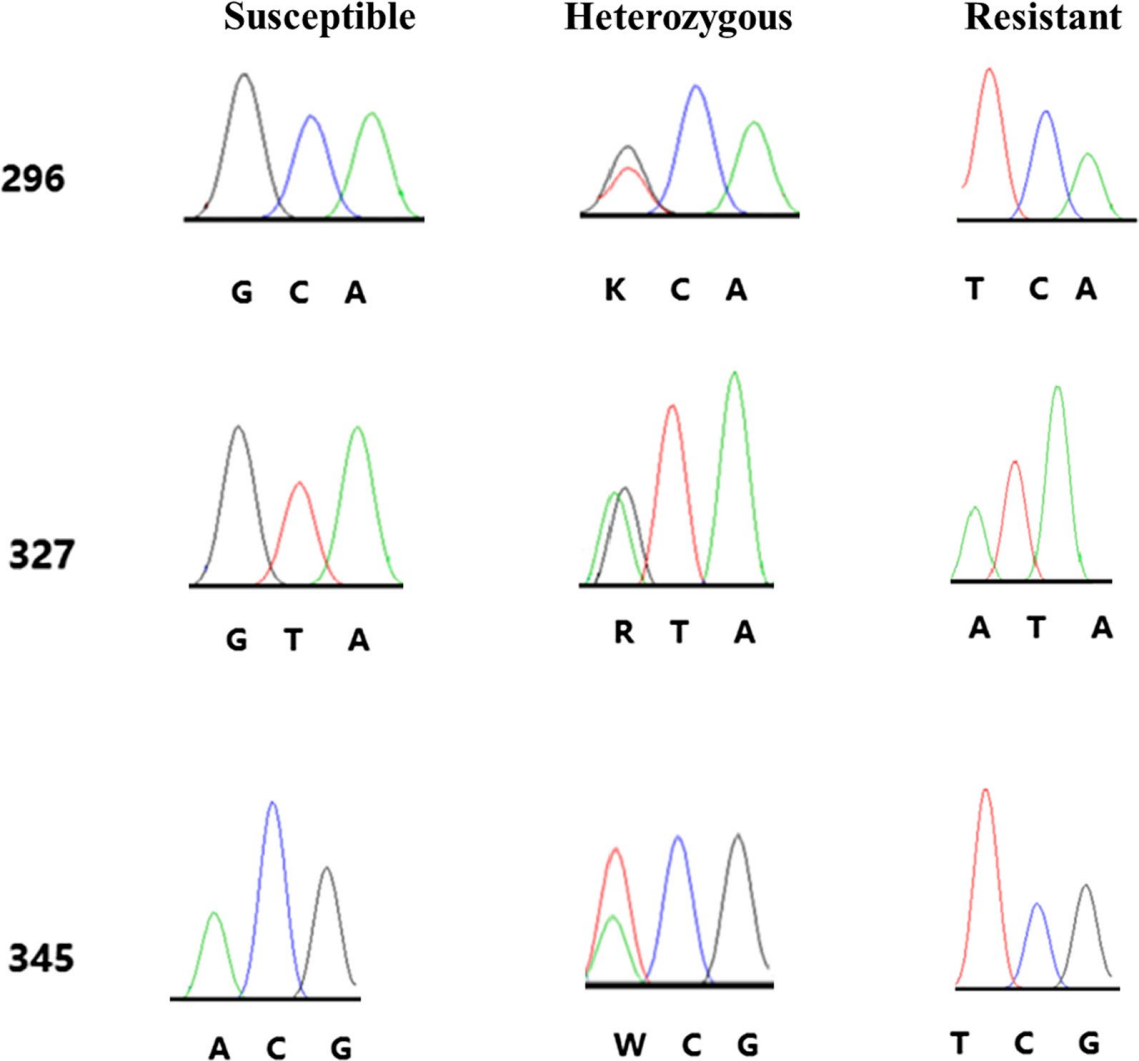
Direct sequencing chromatograph showing three individual genotypes of the three insecticide resistance-related sites

From these sequencing data, 12 non-synonymous polymorphic sites were recorded. Two non-synonymous mutations within exon 7 were identified in the first nucleotide (T/G) of codon 296 and in the first nucleotide (A/G) of codon 327, which lead to A296S and V327I substitutions, respectively (Fig. 5). Ten non-synonymous mutations were observed within exon 8, which result in 345T/S, 349L/M, 356R/G, 357K/L/M/N, 360F/L, 408G/H and 473T/A substitutions, respectively (Fig. 1).

**Fig. 6:**
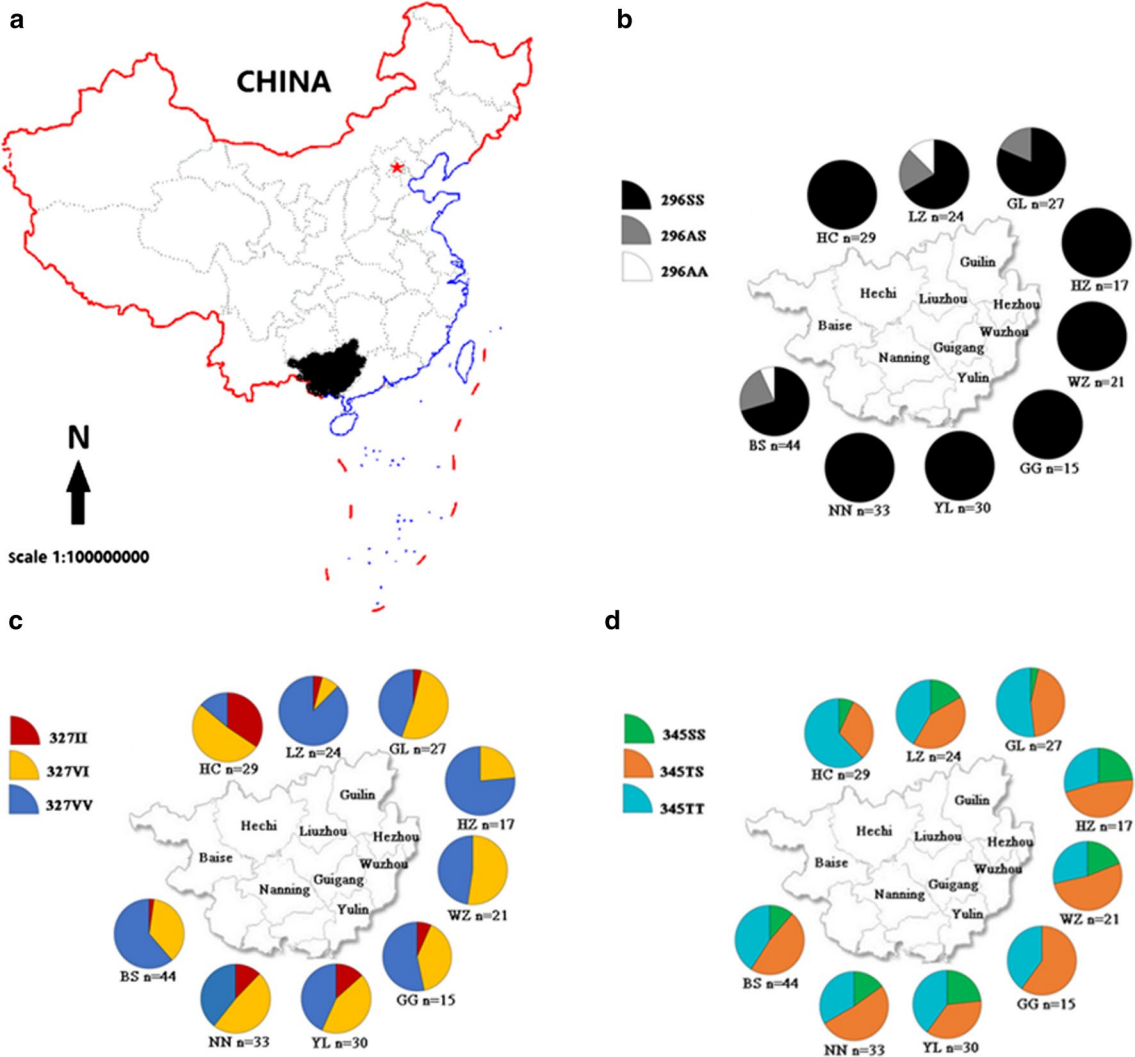
The sampling location (**a**), and distribution frequencies of individual genotypes of *AsRDL* in Guangxi (**b**–**d**). *BS* Baise, *HC* Hechi, *HZ* Hezhou, *GG* Guigang, *GL* Guilin, *LZ* Liuzhou, *NN* Nanning, *WZ* Wuzhou, *YL* Yulin. n represents the sample size

The distribution frequencies of these substitutions were presented in Table 1. Among these substitutions, 356G, 357L, 357N and 408H were detected in only one population at a very low frequency. In contrast, three putative resistance-related substitutions (A296S, V327I and T345S) were widely distributed with relatively higher frequencies (Table 1 and Fig. 6).

**Table 1:** The distribution frequency of the amino acid substitution identified in AsRDL in nine *An. sinensis* populations from Guangxi, China

	296A	296S	327V	327I	345T	345S	349M	349L	356R	356G	357K	357L	357M	357N	360F	360L	408Q	408H	473G	473S
NN		1.000	0.636	0.364	0.591	0.409	0.985	0.015	1.000		0.924		0.076		0.985	0.015	1.000		1.000	
YL		1.000	0.650	0.350	0.583	0.417	0.950	0.050	1.000		0.967		0.033		0.983	0.017	1.000		0.983	0.017
HZ		1.000	0.882	0.118	0.529	0.471	1.000		1.000		0.882		0.088	0.030	1.000		1.000		0.706	0.294
BS	0.182	0.818	0.795	0.205	0.648	0.352	1.000		1.000		0.966	0.011	0.023		0.989	0.011	0.989	0.011	1.000	
WZ		1.000	0.738	0.262	0.548	0.452	0.952	0.048	0.976	0.024	0.976		0.024		1.000		1.000		0.881	0.119
LZ	0.229	0.771	0.917	0.083	0.625	0.375	0.979	0.021	1.000		1.000				1.000		1.000		0.792	0.208
GL	0.093	0.907	0.704	0.296	0.741	0.259	0.944	0.056	1.000		0.907		0.093		1.000		1.000		0.870	0.130
HC		1.000	0.397	0.603	0.776	0.224	0.983	0.017	1.000		0.983		0.017		1.000		1.000		1.000	
GG		1.000	0.733	0.267	0.700	0.300	1.000		1.000		0.933		0.067		1.000		1.000		0.833	0.167

*BS* Baise, *HC* Hechi, *HZ* Hezhou, *GG* Guigang, *GL* Guilin, *LZ* Liuzhou, *NN* Nanning, *WZ* Wuzhou, *YL* Yulin

### Distribution and frequency of resistance-related mutations

The 296S mutation was the most abundant (77–100%), followed by the 345S (22–47%) and 327I (8–60%) mutations. Notably, the resistant allele (296S) had 100% frequency in six of the nine populations (Table 1).

Based on the genotypes of the three resistance-related sites, eleven combinations were observed (Table 2). Most individuals carried at least one resistant mutation (combination 1–10). A few individuals from Baise (6.8%) and Liuzhou (12.5%) were susceptible homozygotes (combination 11). Two combinations (2 and 3) contained mutations in all the three positions. Notably, either 327I or 345S was present only in individuals carrying 296S, and combinations 3, 6 and 7 were distributed in all the nine populations.

**Table 2:** Frequency (in percentage) of combinations of insecticide resistance-associated sites in AsRDL

	Residue sites			Populations								
	296	327	345	NN	YL	HZ	BS	WZ	LZ	GL	HC	GG
Combination 1	*S*/*S*	*I*/*I*	T/T	12.1	13.3		2.3		4.2	3.7	34.5	6.7
Combination 2	*S*/*S*	*I*/V	*S*/*S*				2.3				3.4	
Combination 3	*S*/*S*	*I*/V	T/*S*	36.4	23.3	23.5	11.4	33.3	4.2	22.2	24.1	26.7
Combination 4	*S*/*S*	*I*/V	T/T	12.1	20.0		18.2	19.0		25.9	24.1	13.3
Combination 5	*S*/*S*	V/V	*S*/*S*	15.2	23.3	23.5	9.1	19.0	16.7	3.7	3.4	
Combination 6	*S*/*S*	V/V	T/*S*	15.2	13.3	23.5	25.0	19.0	29.2	18.5	6.9	33.3
Combination 7	*S*/*S*	V/V	T/T	9.1	6.7	29.4	2.3	9.5	12.5	7.4	3.4	20.0
Combination 8	*S*/A	I/V	T/T				4.5		4.2	3.7		
Combination 9	*S*/A	V/V	T/*S*				11.4		8.3	3.7		
Combination 10	*S*/A	V/V	T/T				6.8		8.3	11.1		
Combination 11	A/A	V/V	T/T				6.8		12.5			

The putatively resistant mutations are in italic. The blank cells indicate 0 values

*BS* Baise, *HC* Hechi, *HZ* Hezhou, *GG* Guigang, *GL* Guilin, *LZ* Liuzhou, *NN* Nanning, *WZ* Wuzhou, *YL* Yulin

The distribution frequencies of the *AsRD*L genotypes were showed in Fig. 6. Overall, the distribution showed obvious geographical heterogeneity. At position 296, the heterozygotes were present in Baise, Liuzhou and Guilin, while the susceptible homozygotes were found only in Baise and Liuzhou. Notably, the resistant homzygotes (296SS) was fixed in six of the nine populations. In contrast, the frequencies of resistant homozygotes (327II or 345SS) were much lower. Neither individual homozygous for 327II was detected in Hezhou and Wuzhou, nor mosquito homozygous for 345SS was observed in Guigang (Fig. 6).

### Haplotype diversity of *AsRDL* gene

The sequence data of the 8R fragment that covers both partial intron 7 and exon 8 with a length of 494 bp were used in haplotype analysis in order to obtain more informative result than data from the 7F fragment. From the sequence data of 240 individuals, 38 *AsRDL* haplotypes were identified (Table 3). The 38 haplotypes varied in frequency from 0.2 to 34.8%. Overall five common haplotypes (H1, H2, H3, H17 and H25) accounted for 67% of the haplotype diversity, while sixteen haplotypes were observed only once. The two most common haplotypes (H3 and H17) occurred in all the nine populations.

**Table 3:** Haplotypes of the *AsRDL* gene and their frequencies in nine *An. sinensis* populations from Guangxi, China

Haplotype	Polymorphic sites	Intron type	345aa	NN	YL	HZ	BS	WZ	LZ	GL	HC	GG	Total numbers
H1	ACCCTGATTGAACAAGTCGGCCGGGGGT	[1]	T	4	2	1	4	3	4	3	6	2	29
*H2*	ACCCTGATTGTACAAGTCGGCCGGGGGT	[1]	S	7	9		7	2			3	1	29
H3	ACCCTGCTTGAACAAGTCGGCCGGGGGT	[2]	T	26	28	9	22	13	10	21	28	10	167
H4	ACCCTGCTTGAACAAGTCGGCCGGGGGC	[2]	T				1						1
H5	ACCCTGCTTGAACAAGTCGGCCGGGGAT	[2]	T			3		1	2	3		2	11
H6	ACCCTGCTTGAACAAGTCGGCCGGATGC	[2]	T							1			1
H7	ACCCTGCTTGAACAAGTCGGCCCGATGC	[2]	T				1						1
H8	ACCCTGCTTGAACAAGTCGGTCGGGGGT	[2]	T				1						1
H9	ACCCTGCTTGAACAAGTCGACCGGGGGT	[2]	T						1				1
H10	ACCCTGCTTGAACAAGTGGGCCGGGGGT	[2]	T		1								1
H11	ACCCTGCTTGAACAAGCCGGCCGGGGGT	[2]	T	1			1			1	2		5
H12	ACCCTGCTTGAACAACTCGGCCGGGGGT	[2]	T			1							1
H13	ACCCTGCTTGAACATGTCGGCCGGGGGT	[2]	T	5	2					1	1	1	10
H14	ACCCTGCTTGAACTTGTCGGCCGGGGGT	[2]	T				1						1
H15	ACCCTGCTTGAAGAAGTCGGCCGGGGGT	[2]	T					1					1
H16	ACCCTGCTTGATCAAGTCGGCCGGGGGT	[2]	T	1			1	1	1				4
*H17*	ACCCTGCTTGTACAAGTCGGCCGGGGGT	[2]	S	13	12	2	17	8	2	2	4	3	63
*H18*	ACCCTGCTTGTACAAGTCGGCCGGGGAT	[2]	S		1	7		4	8	4		3	27
*H19*	ACCCTGCTTGTACAAGTCGGCCGGGTGC	[2]	S	1									1
*H20*	ACCCTGCTTGTACAAGTCGGCCGGATGT	[2]	S				1			1			2
*H21*	ACCCTGCTTGTACAAGTCGGCCGGATGC	[2]	S	6	1	1	5	1	1	4	6		25
*H22*	ACCCTGCTTGTTCAAGTCGGCCGGGGGT	[2]	S		1		1						2
H23	ACCCTGCTTAAACAAGTCGGCCGGGGGT	[3]	T			1			2	1		4	8
H24	ACCCTGCTTAAACATGTCGGCCGGGGGT	[3]	T							1		1	2
H25	ACCCCGCACGAACAAGTCGGCCGGGGGT	[4]	T	2			18		5	1	7		33
H26	ACCCCGCACGAACAAGTCGGCCGGAGGT	[4]	T						1				1
H27	ACCCCGCACGAACAAGTCGGCTGGGGGT	[4]	T							1			1
H28	ACCCCGCACGAACAAGTCAGCCGGGGGT	[4]	T					1					1
H29	ACCCCGCACGAACATGTCGGCCGGGGGT	[4]	T			3	1	1		2			7
H30	ACCCCGCACGAACATGTCGGCCGAGGGT	[4]	T				1			1			2
H31	ACCCCGCACGATCAAGTCGGCCGGGGGT	[4]	T		2			1		3	1		7
*H32*	ACCCCGCACGTACAAGTCGGCCGGGGGT	[4]	S		1			2					3
*H33*	ACCCCGCACGTACAAGTCGGCTGGGGGT	[4]	S			6		2	7	3		2	20
H34	ACCTTGCTTGAACAAGTCGGCCGGGGGT	[5]	T				2	1	3				6
H35	ACCTTGCTTGAACAAGTCGGCCGAGGGT	[5]	T				2						2
H36	ACCTTCCTTGAACAAGTCGGCCGGGGGT	[6]	T						1				1
H37	ACACCGCACGAACAAGTCGGCCGGGGGT	[7]	T									1	1
H38	TTCCCGCACGAACAAGTCGGCCGGGGGT	[8]	T				1						1
2N				66	60	34	88	42	48	54	58	30	480

The haplotypes containing the 345S mutation are in italics

*BS* Baise, *HC* Hechi, *HZ* Hezhou, *GG* Guigang, *GL* Guilin, *LZ* Liuzhou, *NN* Nanning, *WZ* Wuzhou, *YL* Yulin

The combination of insecticide resistance-related mutation at position 345 (i.e. 345S) with the other polymorphic sites resulted in 9 resistant haplotypes (H2, H17–22, H32–33) with frequency ranging from 0.2 to 13.1% (Table 3). The nine resistant haplotypes accounted for 36% of the haplotype diversity. Four of them (H2, H17, H18 and H21) were commonly detected (frequency > 5%) and distributed in most of nine populations. The number of resistant haplotypes in each of the nine populations ranged from 3 (Hechi) to 6 (Yulin and Wuzhou).

### Genealogical analysis of *AsRDL* haplotypes

**Fig. 7:**
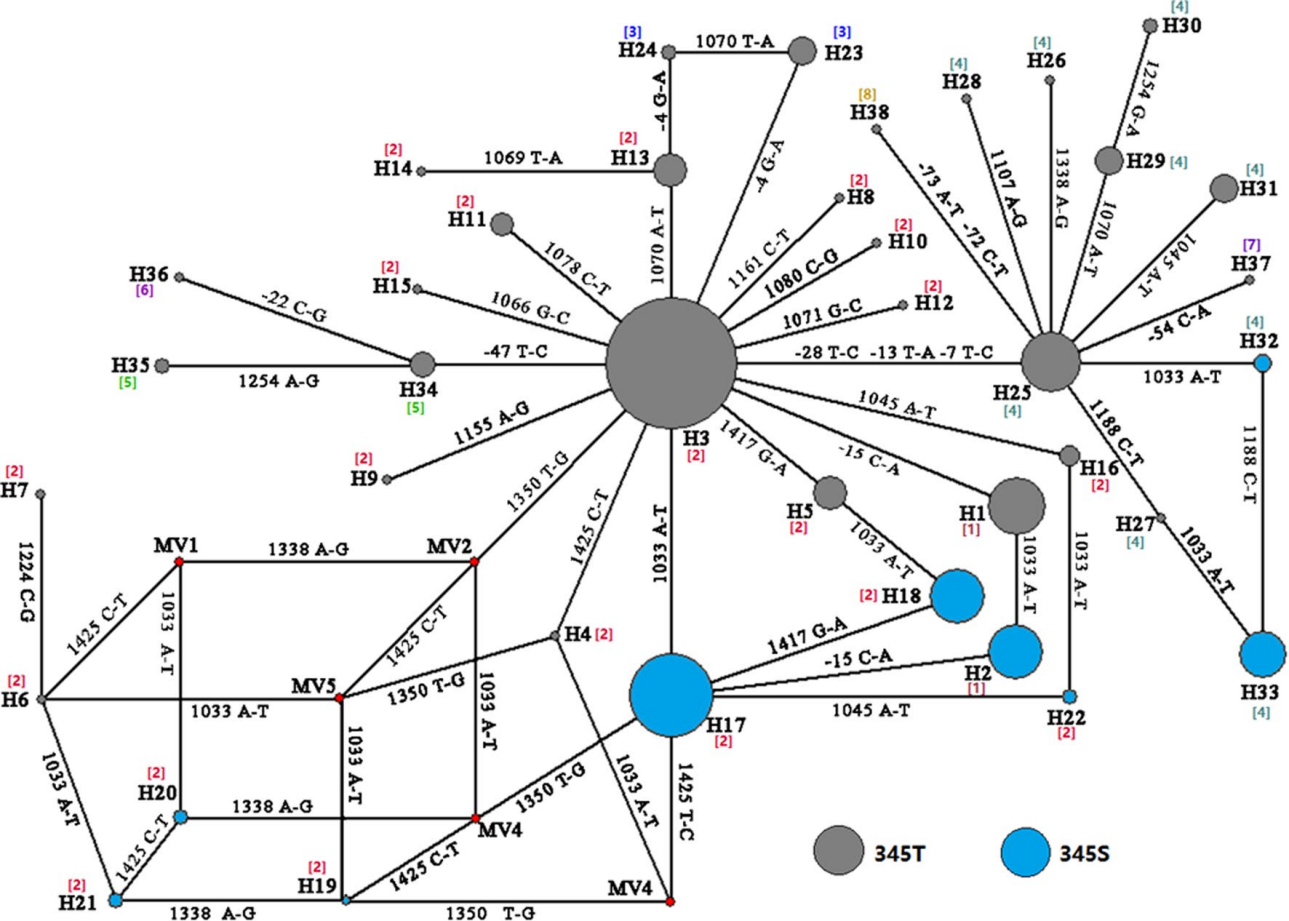
Network showing the genealogy of *AsRDL* haplotypes. The size of each circle is proportional to the corresponding frequency of a certain haplotype identified in Guangxi. Straight line indicates the possible mutational step. The note near the connecting line is referred to the mutation position and base. The intron type for each haplotype is indicated by corresponding number in bracket

**Fig. 8:**
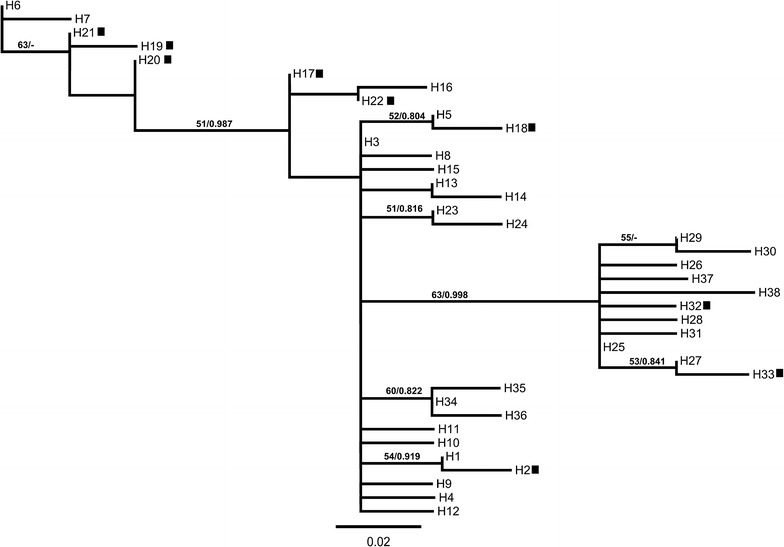
Maximum-likelihood (ML) tree derived from *AsRDL* haplotypes sampled in Guangxi, China. The haplotypes are named by numbers (1–38). Numbers next to nodes of ML tree indicate bootstrap values (%) and Bayesian posterior probabilities, only bootstrap values greater than 50% and Bayesian posterior probabilities greater than 0.8 are shown. Resistant haplotypes are labelled by nearby black squares

The network analysis revealed a complex reticulate genealogical relation of the 38 *AsRDL* haplotypes, and suggested independent mutation events leading to 345S-containing haplotypes (Fig. 7). For example, the resistant H17 was possibly derived from H3, while H32 from H25. Although the phylogenetic tree had lower resolution, the 38 haplotypes were roughly grouped into two major clades (63% bootstrap value) separated by 3 nucleotide substitutions in intron 7 (Fig. 8).

## Discussion

In insects, GABA receptors are ligand-gated chloride channels consisting of presumably the homopentameric RDL subunit encoded by the *RDL* gene. RDLs mediate synapse inhibition in the central nervous system and are important targets for insecticides of widely varied structures such as cyclodienes and fipronil [14]. In this study, the *RDL* gene was identified in *An. sinensis*. The putative AsRDL protein has 557 amino acid residues and shares the common structural features with known Cys-loop ligand-gated chloride channels (Fig. 1). Similar to the finding obtained in *An. funestus* [18], only one GABA-receptor isoform is found in *An. sinensis* in this study.

The RDL subunit has four transmembrane segments (M1, M2, M3 and M4). Point mutations in the M2 and M3 have been documented to confer low sensitivity of the binding site and thus insecticide resistance. In this study, the insecticide resistance associated mutation 296S, and two other mutations (327I and 345S) that have been documented to be linked to 296S [18, 23], were identified and widely distributed in *An. sinensis* populations (Table 1 and Fig. 5). The prediction of the three mutations to be resistance-related is made by reference to other species, thus further functional study is required to confirm it. Whether other mutations (356G, 357L, 357N and 408H) in *An. sinensis*, which are narrowly distributed at a low frequency, contribute to insecticide resistance is unclear.

Point mutations (Ala to Ser/Gly/Asn) in RDL homologues at the site equivalent to 301 in *Drosophila* have been documented to provide resistance to cyclodienes and phenylpyrazoles in several species [17–24, 34]. From the 240 individuals from nine geographical locations, the 296S mutation was detected at a very high frequency. Notably, 100% of individuals in six of the nine populations carried this mutation in the homozygous form. These data indicate a risk of insecticide to cyclodienes and phenylpyrazoles in Guangxi *An. sinensis* populations. However, the GCT to GGT mutation (inducing the A296G change) found in *An. gambiae* [17], and the replacement of Ala with Asn observed in plant hoppers [20, 21], were not detected in *An. sinensis* samples. Given that cyclodienes have now been withdrawn from control programmes, the high frequency of the dieldrin resistance-associated A296S mutation in *An. sinensis* in Guangxi may be the result of the increasing use of insecticides targeting the GABA receptor (e.g. ethiprole) in the agriculture [35].

The 327I mutation which is located in the intracellular loop between TM2 and TM3 has been reported to coexist with 296S in dieldrin-resistant *An. funestus* [18]. Interestingly, a point mutation identical to *An. funestus* (GTA to ATA) was identified in *An. sinensis* in this study (Fig. 5). The impact of the 327I mutation is unknown and worthy of further investigation.

Two mutations at the site 350 in the third membrane spanning domain of RDL have been documented in *Drosophila* [22, 23]. T350 M was observed in fipronil resistant *Drosophila simulans* with Gly301 [22], while T350S was detected in a *Drosophila melanogaster* line (Ral-491) that is homozygous for Ser301 [23]. Interestingly, conserved mutations (A296G + T345 M) were also found in *An. gambiae* [13]. In this study, parallel double mutations (A296S + T345S, corresponding to A301S + T350S/M in *Drosophila*) were detected in *An. sinensis*. This observation lends support to the notion that there is a functional role for mutations at the 345 site [13, 23].

Sequence analyses revealed that *AsRDL* gene had diverse polymorphisms. From the fragment sequences of *AsRDL* from 240 mosquitoes, 38 haplotypes were identified (Table 3). The number and frequency of *AsRDL* haplotypes were different among the nine populations from Guangxi. The most abundant haplotype was H3 (34.8%), followed by H17 (13%). Both H3 and H17 were distributed in all the nine populations tested, and shared the type-2 intron. Similar to the status of *AS*-*VGSC* [12], the high polymorphism of *AsRDL* may be partly explained by their large population size, wide distribution range of *An. sinensis* [36], diverse natural landscapes and different local insecticide selective pressure in Guangxi.

The genealogical relation and phylogenetic tree analyses support two independent origins of the resistance related 345S mutation in AsRDL (Figs. 7, 8). The two most common susceptible haplotypes (H3 and H25) could be considered as possible ancestors of resistant haplotypes respectively. The six resistant haplotypes (H17–22) were derived from their common susceptible ancestor H3 with one to four mutational steps, whereas the other two resistant haplotypes (H32 and H33) were the result of one and two mutational steps respectively from H25.

## Conclusions

Three insecticide resistance related mutations (296S, 327I and 345S) in AsRDL were detected in Guangxi. The extremely high frequency or fixation of the 296S mutation, and the occurrence of 327I and 345S in addition to 296S, in all populations tested strongly indicate a risk of multiple and/or cross resistance to cyclodienes (e.g. dieldrin and α-endosulfan) and phenylpyrazole (e.g. fipronil and ethiprole). The haplotype diversity plus genetic heterogeneities in the geographical distribution, and multiple origins of *AsRDL* alleles call for a location-customized strategy for monitoring and management of insecticide resistance.

## Abbreviations

*RDL*: resistant to dieldrin
Guangxi: Guangxi Zhuang Autonomous Region
GABA: gamma-aminobutyric acid
